# miR-370 Sensitizes TMZ Response Dependent of MGMT Status in Primary Central Nervous System Lymphoma

**DOI:** 10.1007/s12253-019-00605-4

**Published:** 2019-02-02

**Authors:** Xinwei Li, Xueying Xu, Keng Chen, Haijian Wu, Yirong Wang, Shuxu Yang, Kun Wang

**Affiliations:** 1 Department of Neurosurgery, Sir Run Run Shaw Hospital, Medical College, Zhejiang University, Hangzhou, 310016 People’s Republic of China; 2 Department of Neurosurgery, Hangzhou Xiasha Hospital, Sir Run Run Shaw Hospital, Medical College, Zhejiang University, Hangzhou, 310016 People’s Republic of China

**Keywords:** Primary central nervous system lymphoma, miR-370, MGMT, Temozolomide, Drug resistance

## Abstract

Primary central nervous system lymphoma (PCNSL) is an aggressive and rare subtype of non-Hodgkin lymphoma, arising exclusively in the CNS with a poor prognosis. Previous evidence has proved that MGMT was a promising target involving in TMZ resistance of PCNSL. Our study described a new miR-370-mediated mechanism of MGMT regulation in PCNSL. We first showed that miR-370 was downregulated in PCNSL tissues, while MGMT was inversely overexpressed. It was also observed that miR-370 suppressed the expression of MGMT. Additionally, upregulation of miR-370 significantly increased TMZ sensitivity dependent of MGMT, thus suppressed Raji cell proliferation and induced apoptosis in vitro. In conclusion, these results suggest that miR-370 is a potential target in PCNSL treatment.

## Introduction

Primary central nervous system lymphomas (PCNSLs) are rare non-Hodgkin lymphomas only confined to the central nervous system, such as brain, eyes, leptomeninges, or spinal cord, which account for 2 to 3% of all primary intracranial tumours [1, 2]. Diffuse large B cell lymphoma (DLBCL) is one of the most common types of the PCNSL. Age has been demonstrated as a significant prognostic factor in PCNSL patients [3]. PCNSL is much more sensitive to initial aggressive radiotherapy and chemotherapy, including high-dose based methotrexate, followed by whole-brain irradiation [4–6], compared to the other malignant CNS tumors. But the best therapeutic approach remains uncertain, due to the trouble of drug delivery across the blood-brain barrier [7]. In addition, the neurotoxicity, delayed diagnosis and treatment lead to final relapse of PCNSL. Thus the overall survival for PCNSL remains poor for most patients [8].

Temozolomide with good CNS penetration and low toxicity is an oral alkylating agent that affects O6-methylguanine-DNA-methyltransferase (MGMT) status, which are also proved to be effective against non-Hodgkin’s lymphoma [9, 10]. Recently the usage of temozolomide as salvage therapy has been reported to treat the patients with refractory or relapsed PCNSL [11–13]. Temozolomide is also a good choice for the elderly patients with impaired renal function or other comorbidities, and can’t receive HD-MTX. As MGMT gene promoter methylation and high expression are promising predictors in TMZ-treated glioblastoma patients, we further determined the MGMT status involving in the PCNSL patients.

MicroRNAs (miRNAs) are small non-coding RNAs that post-transcriptionally downregulate gene expression via binding to target mRNA [14, 15], thus can function as tumour suppressors or oncogenes through downstream extensive network of signaling pathway [16]. Recent years, miRNAs have gained increasing attention from researchers due to their crucial functions in many cellular affairs, and also the potential to serve as clinical targets and markers. Though evidences have revealed the dysregulation of miRNAs in PCNSL, the understanding of functional roles of these abnormally expressed miRNAs are still limited.

In the present study, we sought to explore the association between miR-370 and MGMT, provide evidence that miR-370 regulates MGMT expression in PCNSL cells, further to increase the TMZ sensitivity.

## Materials and Methods

### Cell Culture, Plasmid Construction and Transfection

The Raji Burkitt’s lymphoma cell line was purchased from Type Culture Collection of the Chinese Academy of Sciences, Shanghai, China, and cultured in RPMI 1640 (Sigma) containing 10% fetal bovine serum. The cells were incubated at 37 °C in a humidified atmosphere of 5% CO2 in air. The pEGFP MGMT overexpression plasmid, MGMT siRNA, the scramble, miR-370 mimics and miR-370 inhibitors were constructed by GeneChem Co., Ltd. (Suzhou, China).

Raji cells were first seeded in 24-well plates. 24 h later, MGMT overexpression plasmid, MGMT siRNA, the scramble, miR-370 mimics or miR-370 inhibitors in 50 μL of medium was respectively mixed with 2 μL of Lipofectamine 2000 (Invitrogen, USA) dissolved in 50 μL of RPMI and then placed at room temperature for 20 min. The mixed 100-μL solutions were then added to each well containing 400 μL of medium. And cells were harvested for the next step.

### Human PCNSL Samples

Twenty human PCNSL samples and twenty controls (cervical lymph nodes tissues) were obtained from the Department of Neurosurgery, Sir Run Run Shaw Hospital, Medical College, Zhejiang University, China. Informed consents were obtained from patients who had received a diagnosis of PCNSLs. Samples were gained by resection or biopsy, and immediately frozen in liquid nitrogen for subsequent total RNA extraction.

### RNA Extraction and Quantitative Real-Time Polymerase Chain Reaction

Total RNA was isolated from, human PCNSL specimens, nodal tissues or raji cells with TRIzol reagent (Invitrogen). The process quantitative of real-time polymerase chain reaction (qRT-PCR) was performed according to the manufacturer’s instructions [17].

### Immunohistochemistry and Western Blotting

Immunohistochemistry and Western blot were performed as previously described [15].

### MTT Assay

Transfected Raji cells were gained and seeded in 96-well assay plates. After 12, 24 and 48 h later, 3-(4, 5-dimethylthiazol-2-yl)-2, 5- diphenyltetrazolium bromide (MTT) (Sigma, USA) was added, and the cells were incubated at 37 °C. 4 h later, dimethyl sulfoxide (Sigma) was used to dissolve the formazan crystals. Optical density was measured at the wavelength of 570 nm. The data are obtained for the analysis.

### Flow Cytometry Analysis

The transfected Raji cells were incubated for 12, 24 and 48 h, then the cells were collected. After being washed with pre-chilled phosphate buffered saline (PBS) twice, the Raji cells were stained with FITC-labeled Annexin V and propidium iodide. FC-50 flow cytometry (Beckman Coulter) was used to assess the cell apoptosis.

### Statistical Analysis

Statistics were performed using the SPSS version 11.0. Data were expressed as the mean ± standard deviation. Student’s t test or analysis of variance was used to evaluate the comparisons between groups. All differences were considered to be statistically significant at the level of *P* < 0.05.

## Results

### miR-370 and MGMT Expression in PCNSL Samples

**Fig. 1:**
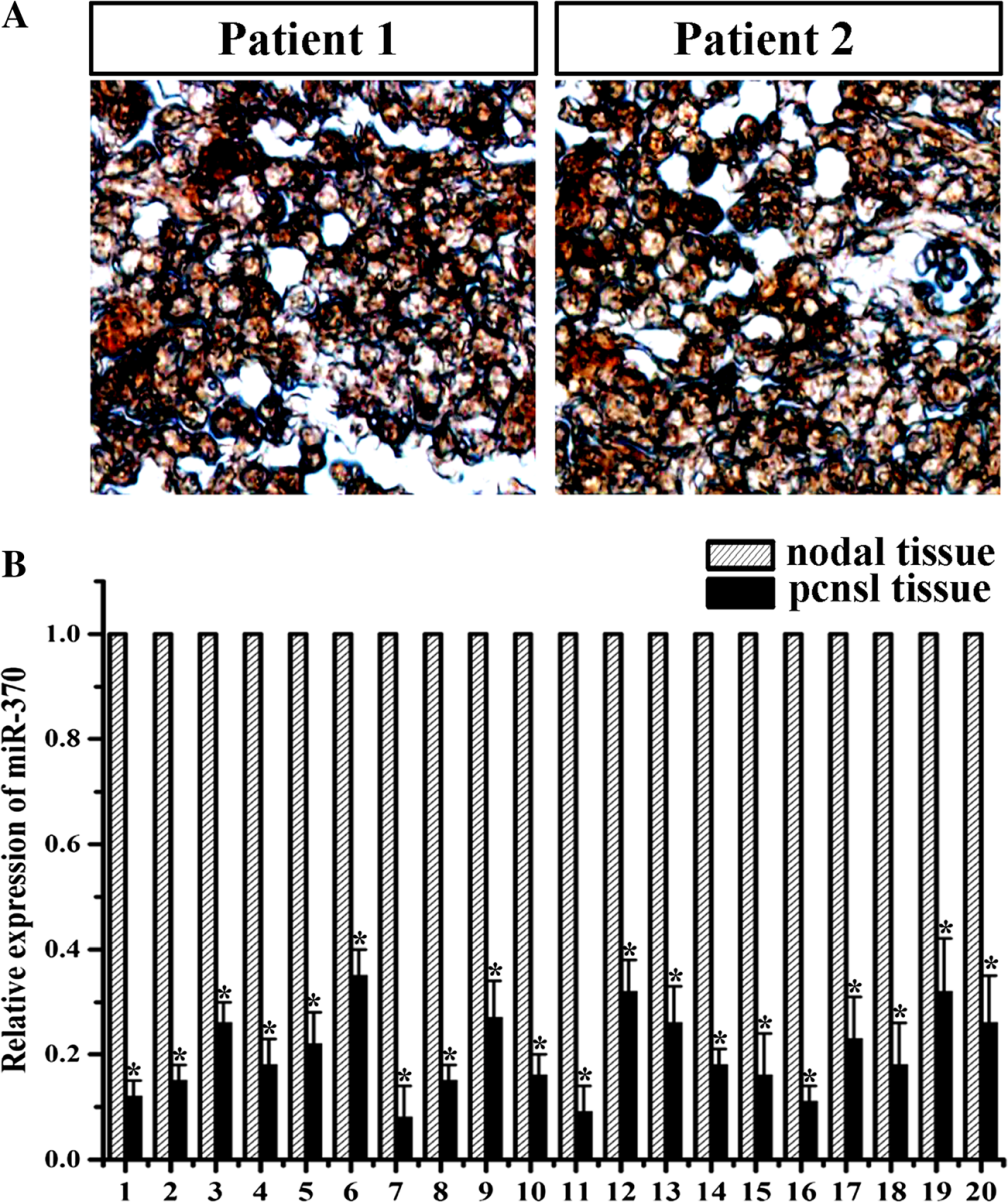
Expression of MGMT and miR-370 were detected in PCNSL and nodal tissues. **a** Immunohistochemistry revealed high expression of MGMT in twenty clinical specimens. **b** qRT-PCR results showed that miR-370 was differentially downregulated in PCNSL tissues compared to nodal tissues

To determine the expression of MGMT in PCNSL tissues, the immunostainings for MGMT were examined in eight PCNSL cases. It was shown in cell nuclei with an immunoreactivity of high intensity in all cases (Fig. 1a). Total RNAs were further extracted from twenty PCNSL and twenty nodal samples. The expression levels of miR-370 were analyzed by qRT-PCR. As shown in Fig. 1b, the levels of miR-370 expression in PCNSL tissues were significantly decreased, compared with nodal tissues. Taken together, our results revealed that miR-370 and MGMT were inversely expressed in human PCNSL samples.

### miR-370 Regulates MGMT Expression

**Fig. 2:**
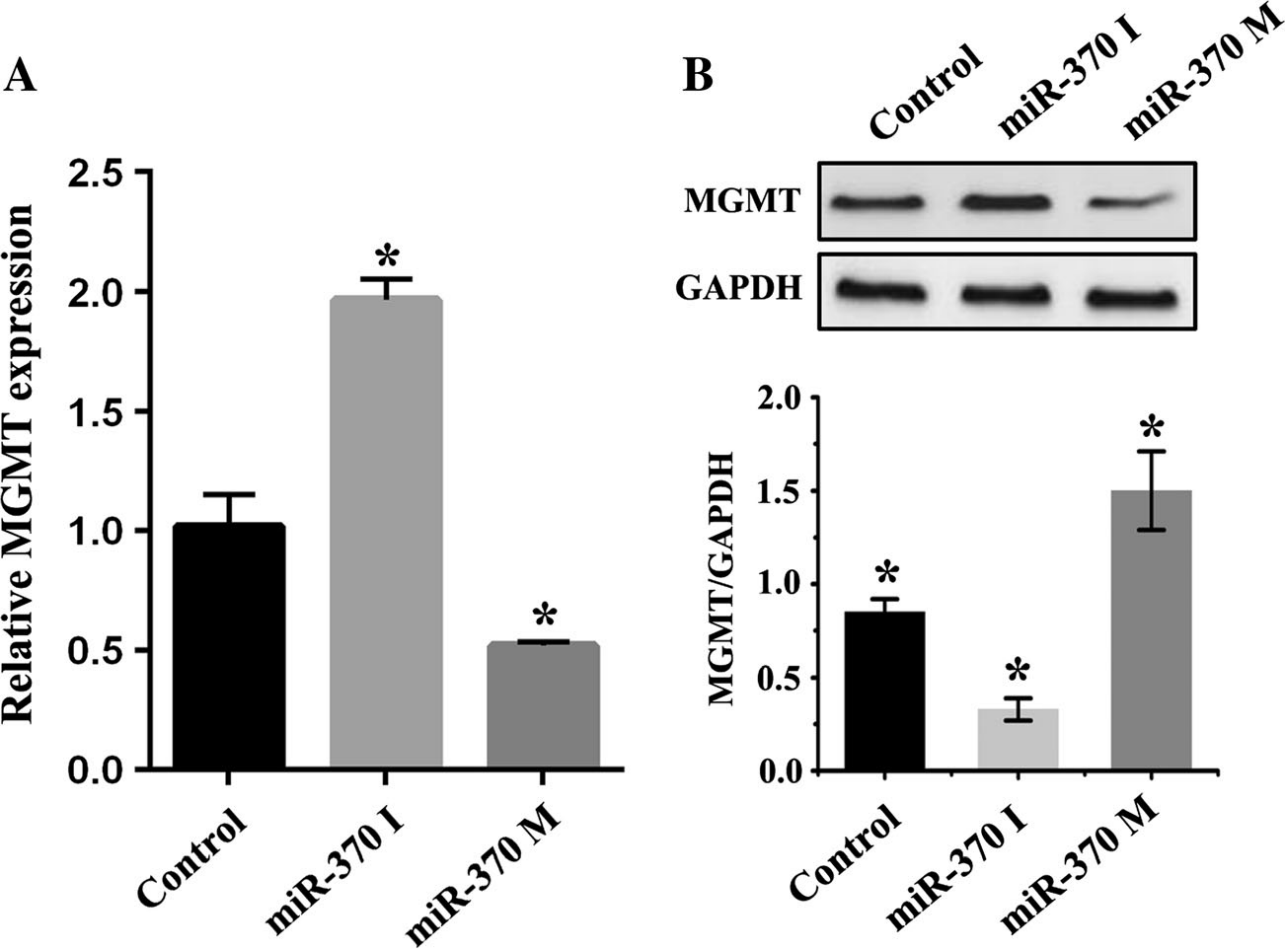
miR-370 regulates MGMT expression in raji cells. **a** qRT-PCR demonstrated that miR-370 inhibitors increased expression of MGMT at mRNA level, while miR-370 mimics suppressed MGMT mRNA expression. **b** The result of MGMT protein level examined by western blot was similar with (**a**) after being transfected with miR-370 inhibitors or mimics

We next explored the correlation between miR-370 and MGMT in vitro. Using bioinformatic methods, we found that MGMT was a potential target of miR-370. As shown in Fig. 2, miR-370 inhibitors or mimics were transfected into raji cells, and 48 h later the transfected cells were harvested. Results revealed that miR-370 inhibitors suppressed both expression of MGMT mRNA and protein, while miR-370 mimics upregulated MGMT expression. Together, these results provide evidence that miR-370 regulates MGMT expression at both mRNA and post-transcriptional level.

### miR-370 Affects PCNSL Cell Proliferation and Apoptosis Via MGMT

**Fig. 3:**
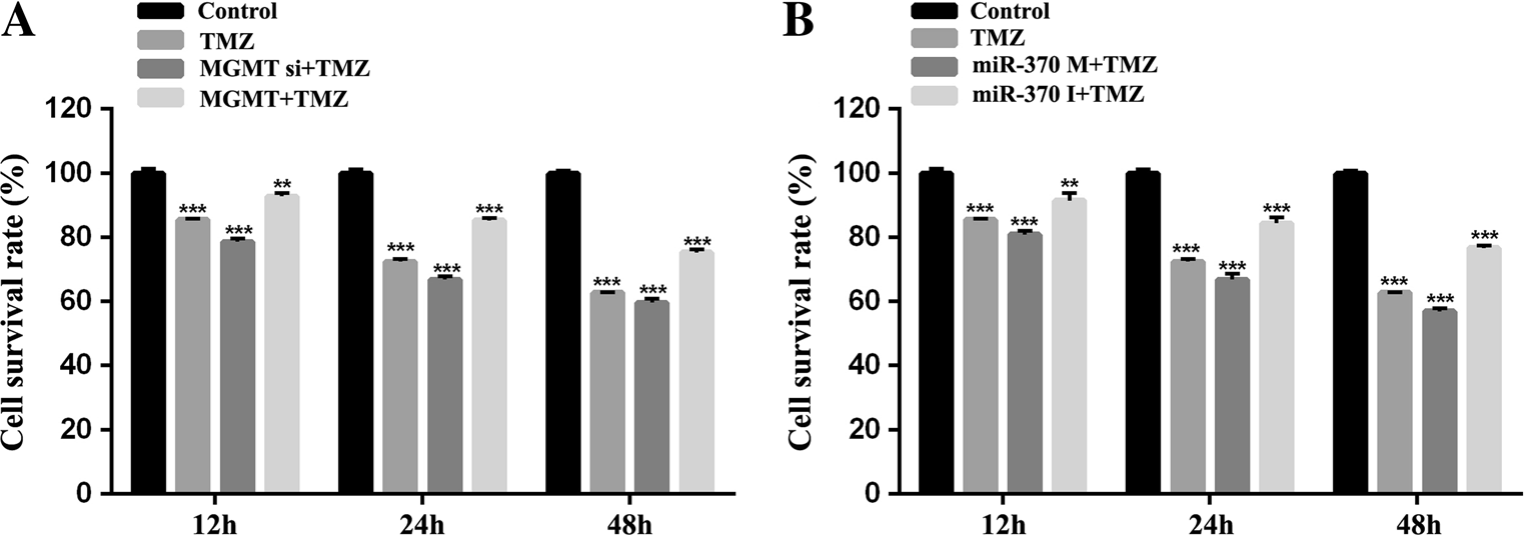
Effect of miR-370 and MGMT on the TMZ sensitivity and raji cell proliferation. **a** TMZ obviously suppressed raji cell proliferation at 12 h, 24 h and 48 h. MGMT siRNA increased the TMZ sensitivity to cell proliferation, while MGMT lead to TMZ resistance. **b** miR-370 mimics were shown to inhibit cell proliferative ability, and enhance the TMZ sensitivity, while suppression of miR-370 caused TMZ resistance

**Fig. 4:**
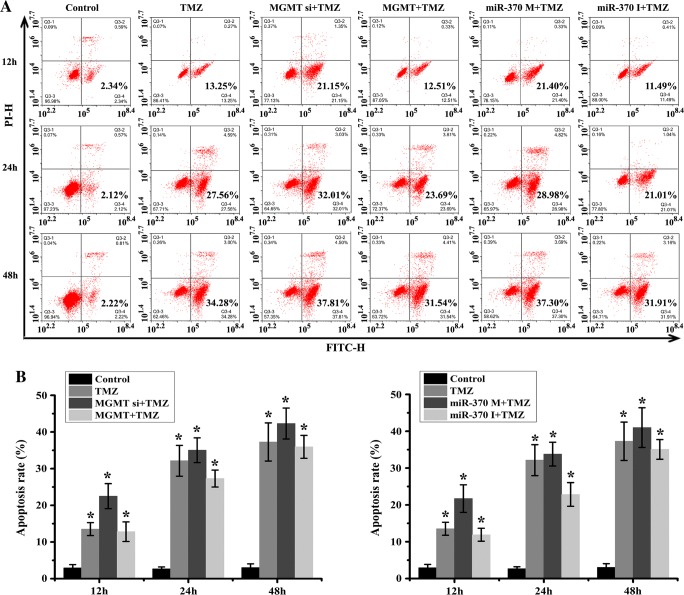
Effect of miR-370 and MGMT on the TMZ sensitivity and raji cell apoptosis. **a** TMZ induced raji cell apoptosis at 12 h, 24 h and 48 h. MGMT siRNA augment the effect of TMZ on cell apoptosis, while MGMT decreased TMZ sensitivity. **b** upregulation of miR-370 tended to cause TMZ hypersensitivity on raji cells, while miR-370 inhibitors lead to TMZ hyposensitivity

**Fig. 5:**
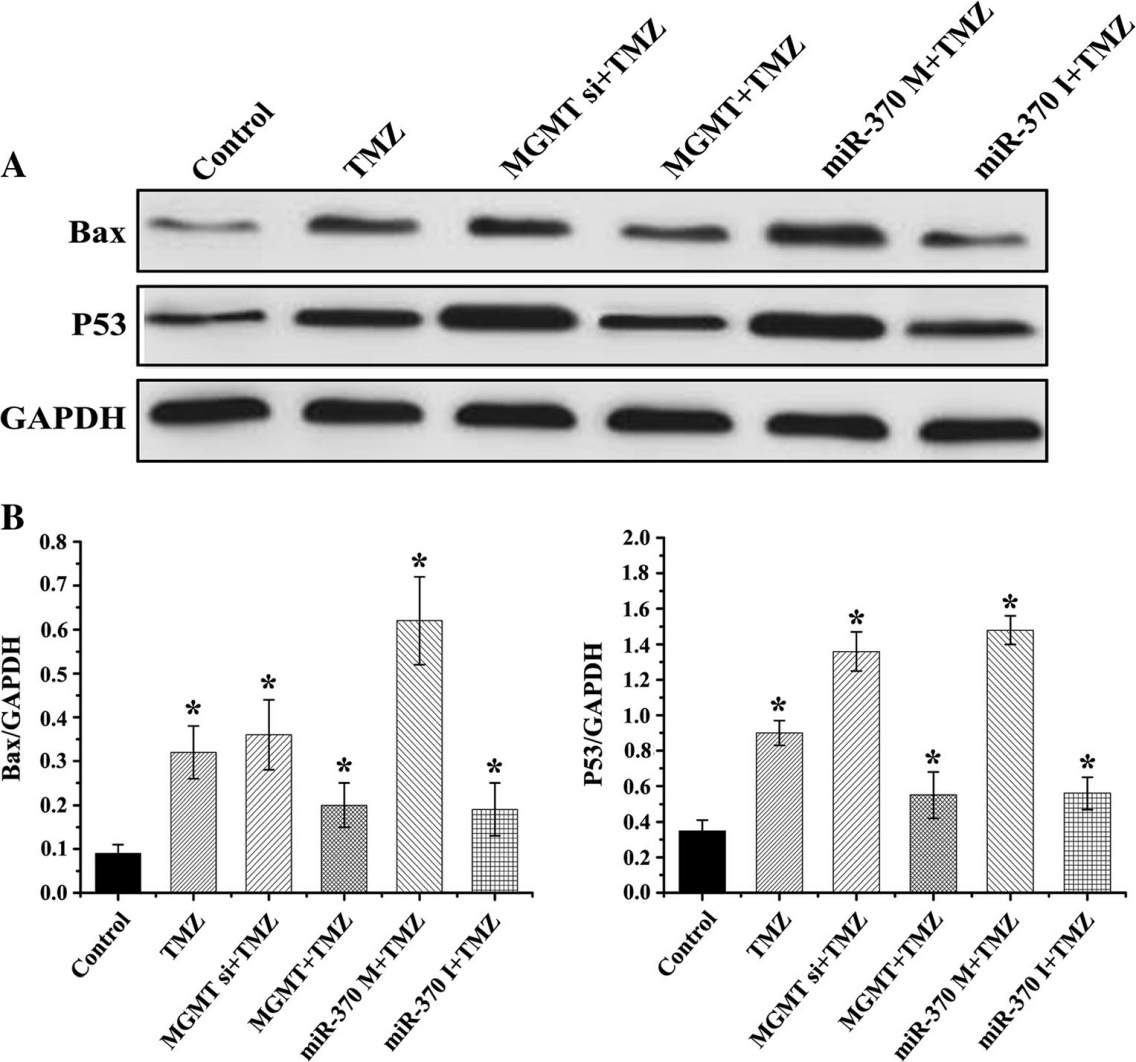
miR-370 affected PCNSL increased apoptotic cell death via MGMT Expression of apoptosis-related markers detected by miR-370 and MGMT treatment. TMZ increased Bax and P53 expression. Bax and P53 were upregulated in MGMT si + TMZ and miR-370 M group. MGMT and miR-370 I decreased TMZ effect on apoptosis-related markers.

To confirm the modulation of miR-370 on TMZ sensitivity, we divided six groups in the experiment, including Control, TMZ, MGMT+TMZ, MGMT siRNA+TMZ, miR-370 mimics+TMZ and miR-370 inhibitor+TMZ groups. As shown in Figs. 3 and 4, TMZ obviously suppressed raji cell proliferation ability and induced cell death. Further we found that MGMT siRNA transfection increased the TMZ sensitivity to raji cells, while MGMT caused TMZ resistance. The similar results were also confirmed in miR-370 mimics+TMZ and miR-370 inhibitor+TMZ groups. Furthermore, the protein expression of apoptosis-related molecules (P53 and Bax) was consistent with the previous study (Fig. 5). In conclusion, TMZ sensitivity consistently correlated with the overexpression of miR-370 and inhibition of MGMT; miR-370 affected PCNSL cell proliferation and increased apoptosis via MGMT.

## Discussion

Though a number of evidences have supported that high-dose methotrexate (MTX)-based chemotherapy is the first-line therapy for patients with primary CNS lymphoma (PCNSL), the poor outcome still can’t be improved for the majority of patients due to the reason of delayed neurotoxicity and renal dysfunction [18]. Because of well-toleration and good penetration of blood brain barrier, temozolomide has recently emerged as an important choice for PCNSL patients in elderly or salvage therapy after failure to high-dose MTX [19–21], or in combination with rituximab and methotrexate [10, 22, 23]. However current TMZ therapy is significantly impeded by TMZ resistance, in which MGMT was activated to repair DNA changes caused by these agents in PCNSL cells.

Dysregulation of miR-370 has been described in tumorigenesis and progression by functioning as a tumor suppressor gene. Wu et al. reported that miR-370 was down-regulated in human laryngeal squamous cell carcinoma tissues, and inhibited cell proliferation in LSCC through downregulation of FoxM1 [24]. It was additionally demonstrated that expression of miR-370 was decreased in endometrioid ovarian cancer cells, and miR-370 suppressed proliferation and promotes endometrioid ovarian cancer chemosensitivity to cDDP by negatively regulating ENG [25]. miR-370 was also revealed to function as tumor suppressor gene in the gastric carcinoma and acute myeloid leukemia [26, 27]. Researchers have shown that miRNAs were abnormally expressed in PCNSL, many of which were implicated in various molecular functions, involving in the network of signaling, such as Myc pathway, terminal B cell differentiation, and inflammatory cytokines, et al [28–32], but little was referred to drug resistance of PCNSL. In the glioblastoma multiforme, it was found that miRNA-370-3p affected the sensitivity of GBM cells to TMZ via MGMT expression, which was not identified in the PCNSL [33].

In the present work, our results have revealed that miR-370 was significantly down-regulated in PCNSL samples compared with the nodal tissues. Furthermore, MTT assay and apoptosis assay supported our hypothesis that ectopic overexpression of miR-370 enhanced the cytotoxic effect of TMZ on raji cells, caused cell proliferation suppression and induced apoptosis. Furthermore, miR-370-overexpressing cells exhibited an increased expression of apoptotic markers upon TMZ treatment, such as P53 and Bax. As MGMT participated in the repair of DNA, miR-370 may induce MGMT downregulation, and render the raji cells unable to repair DNA damage, finally lead to cell death. Collectively, our data suggested that miR-370 plays a vital role in TMZ resistance of raji cells via MGMT.

In conclusion, miR-370 was detected for the first time to be downregulated in PCNSL tissues, significantly correlated with the TMZ resistance and functioned as a tumor suppressor in raji cells depending on MGMT status. However, animal models, larger human PCNSL samples, and other studies, are required to validate the results from the present study.

## References

[1] Batchelor T, Loeffler JS (2006). Primary CNS lymphoma. J Clin Oncol.

[2] Korfel A, Schlegel U (2013). Diagnosis and treatment of primary CNS lymphoma. Nat Rev Neurol.

[3] Olson JE, Janney CA, Rao RD, Cerhan JR, Kurtin PJ, Schiff D, Kaplan RS, O'Neill BP (2002). The continuing increase in the incidence of primary central nervous system non-Hodgkin lymphoma: a surveillance, epidemiology, and end results analysis. Cancer.

[4] Ferreri AJ, Verona C, Politi LS, Chiara A, Perna L, Villa E, Reni M (2011). Consolidation radiotherapy in primary central nervous system lymphomas: impact on outcome of different fields and doses in patients in complete remission after upfront chemotherapy. Int J Radiat Oncol Biol Phys.

[5] Moise L, Matta C, Hanna C, Pilorge S, Fermé C, Durrbach A, Ribrag V (2011). Methotrexate- and/or cytarabine-based chemotherapy may be effective and safe in solid-organ transplant recipients with primary central nervous system lymphomas. Leuk Lymphoma.

[6] Shi X, Zhang X, Yi C, Wang X, Chen Z, Zhang B (2013). The combination of 13N-ammonia and 18F-FDG in predicting primary central nervous system lymphomas in immunocompetent patients. Clin Nucl Med.

[7] Mead GM, Bleehen NM, Gregor A, Bullimore J, Shirley D, Rampling RP, Trevor J, Glaser MG, Lantos P, Ironside JW, Moss TH, Brada M, Whaley JB, Stenning SP (2000). A medical research council randomized trial in patients with primary cerebral non-Hodgkin lymphoma: cerebral radiotherapy with and without cyclophosphamide, doxorubicin, vincristine, and prednisone chemotherapy. Cancer.

[8] Panageas KS, Elkin EB, DeAngelis LM, Ben-Porat L, Abrey LE (2005). Trends in survival from primary central nervous system lymphoma, 1975–1999: a population-based analysis. Cancer.

[9] Herrlinger U, Kuker W, Platten M (2002). First-line therapy with temozolomide induces regression of primary CNS lymphoma. Neurology.

[10] Reni M, Mason W, Zaja F, Perry J, Franceschi E, Bernardi D, Dell'Oro S, Stelitano C, Candela M, Abbadessa A, Pace A, Bordonaro R, Latte G, Villa E, Ferreri AJ (2004). Salvage chemotherapy with temozolomide in primary CNS lymphomas: preliminary results of a phase II trial. Eur J Cancer.

[11] Reni M, Ferreri AJ, Landoni C, Villa E (2000). Salvage therapy with temozolomide in an immunocompetent patient with primary brain lymphoma. J Natl Cancer Inst.

[12] Strik HM, Spreer A, Nagel H, Jacob S, Jung W, Kitze B, Bähr M (2004). Clinical response following adjuvant temozolomide in a patient with primary cerebral lymphoma. Anticancer Res.

[13] Reni M, Zaja F, Mason W, Perry J, Mazza E, Spina M, Bordonaro R, Ilariucci F, Faedi M, Corazzelli G, Manno P, Franceschi E, Pace A, Candela M, Abbadessa A, Stelitano C, Latte G, Ferreri AJ (2007). Temozolomide as salvage treatment in primary brain lymphomas. Br J Cancer.

[14] Wang K, Xie D, Xie J, Wan Y, Ma L, Qi X, Yang S (2015). MiR-27a regulates Wnt/beta-catenin signaling through targeting SFRP1 in glioma. Neuroreport.

[15] Wang K, Wang X, Zou J, Zhang A, Wan Y, Pu P, Song Z, Qian C, Chen Y, Yang S, Wang Y (2013). miR-92b controls glioma proliferation and invasion through regulating Wnt/beta-catenin signaling via nemo-like kinase. Neuro-Oncology.

[16] Yang S, Wang K, Qian C, Song Z, Pu P, Zhang A, Wang W, Niu H, Li X, Qi X, Zhu Y, Wang Y (2012). A predicted miR-27a-mediated network identifies a signature of glioma. Oncol Rep.

[17] Nass D, Rosenwald S, Meiri E, Gilad S, Tabibian-Keissar H, Schlosberg A, Kuker H, Sion-Vardy N, Tobar A, Kharenko O, Sitbon E, Lithwick Yanai G, Elyakim E, Cholakh H, Gibori H, Spector Y, Bentwich Z, Barshack I, Rosenfeld N (2009). MiR-92b and miR-9/9* are specifically expressed in brain primary tumors and can be used to differentiate primary from metastatic brain tumors. Brain Pathol.

[18] DeAngelis LM (2015). Neuro-oncology: primary CNS lymphoma treatment-the devil is in the details. Nat Rev Neurol.

[19] Omuro AM, Taillandier L, Chinot O, Carnin C, Barrie M, Hoang-Xuan K (2007). Temozolomide and methotrexate for primary central nervous system lymphoma in the elderly. J Neuro-Oncol.

[20] Reni M, Mazza E, Foppoli M, Ferreri AJ (2007). Primary central nervous system lymphomas: salvage treatment after failure to high dose methotrexate. Cancer Lett.

[21] Kurzwelly D, Glas M, Roth P, Weimann E, Lohner H, Waha A, Schabet M, Reifenberger G, Weller M, Herrlinger U (2010). Primary CNS lymphoma in the elderly: temozolomide therapy and MGMT status. J Neuro-Oncol.

[22] Enting RH, Demopoulos A, DeAngelis LM, Abrey LE (2004). Salvage therapy for primary CNS lymphoma with a combination of rituximab and temozolomide. Neurology.

[23] Omuro AM, Taillandier L, Chinot O, Carnin C, Barrie M, Hoang-Xuan K (2007). Temozolomide and methotrexate for primary central nervous system lymphoma in the elderly. J Neuro-Oncol.

[24] Yungang W, Xiaoyu L, Pang T, Wenming L, Pan X (2014). miR-370 targeted FoxM1 functions as a tumor suppressor in laryngeal squamous cell carcinoma (LSCC). Biomed Pharmacother.

[25] Chen XP, Chen YG, Lan JY, Shen ZJ (2014). MicroRNA-370 suppresses proliferation and promotes endometrioid ovarian cancer chemosensitivity to cDDP by negatively regulating ENG. Cancer Lett.

[26] Feng Y, Wang L, Zeng J, Shen L, Liang X, Yu H, Liu S, Liu Z, Sun Y, Li W, Chen C, Jia J (2013). FoxM1 is overexpressed in helicobacter pylori-induced gastric carcinogenesis and is negatively regulated by miR-370. Mol Cancer Res.

[27] Zhang X, Zeng J, Zhou M, Li B, Zhang Y, Huang T, Wang L, Jia J, Chen C (2012). The tumor suppressive role of miRNA-370 by targeting FoxM1 in acute myeloid leukemia. Mol Cancer.

[28] Fischer L, Hummel M, Korfel A, Lenze D, Joehrens K, Thiel E (2011). Differential micro-RNA expression in primary CNS and nodal diffuse large B-cell lymphomas. Neuro-Oncology.

[29] Zheng J, Xu J, Ma S, Sun X, Geng M, Wang L (2013). Clinicopathological study of gene rearrangement and microRNA expression of primary central nervous system diffuse large B-cell lymphomas. Int J Clin Exp Pathol.

[30] Robertus JL, Harms G, Blokzijl T, Booman M, de Jong D, van Imhoff G, Rosati S, Schuuring E, Kluin P, van den Berg A (2009). Specific expression of miR-17-5p and miR-127 in testicular and central nervous system diffuse large B-cell lymphoma. Mod Pathol.

[31] Sun G, Hou YB, Jia HY, Bi XH, Yu L, Chen DJ (2016). MiR-370 promotes cell death of liver cancer cells by Akt/FoxO3a signalling pathway. Eur Rev Med Pharmacol Sci.

[32] Cao X, Liu D, Yan X, Zhang Y, Yuan L, Zhang T, Fu M, Zhou Y, Wang J (2013). Stat3 inhibits WTX expression through up-regulation of microRNA-370 in Wilms tumor. FEBS Lett.

[33] Gao Y, Chen X, Liu H (2016) Up-regulation of miR-370-3p restores glioblastoma multiforme sensitivity to temozolomide by influencing MGMT expression. Sci Rep 6:32972 10.1038/srep32972 PMC5011744 27595933

